# Time trends in self-reported depressive symptoms, prescription of antidepressants, sedatives and hypnotics and the emergence of social media among Norwegian adolescents

**DOI:** 10.1371/journal.pone.0295384

**Published:** 2023-12-27

**Authors:** Lars Lien, Tore Bonsaksen, Tonje Holte Stea, Annette Løvheim Kleppang, Anne Mari Steigen, Marja Leonhardt

**Affiliations:** 1 Faculty of Social and Health Sciences, Inland Norway University of Applied Sciences, Elverum, Norway; 2 Norwegian National Advisory Unit on Concurrent Substance Abuse and Mental Health Disorders, Innlandet Hospital Trust, Brumunddal, Norway; 3 Department of Health, Faculty of Health Studies, VID Specialized University, Stavanger, Norway; 4 Department of Health and Nursing Science, University of Agder, Kristiansand, Norway; 5 Faculty of Health Studies, VID Specialized University, Oslo, Norway; Tribhuvan University Institute of Medicine, NEPAL

## Abstract

**Background:**

Research has shown increased mental health problems and use of prescription drugs among adolescents in recent years and social media use has been linked to poorer mental health. However, trend studies concerning these topics are scarce. The purpose of this study was to analyze gender-specific trends in a) symptoms of depression and loneliness, and b) prescription of antidepressants, hypnotics and sedatives, in relation to the emergence of social media among adolescents in Norway.

**Methods:**

This is an ecological study using data from the ‘Young in Oslo’ surveys from 1996 to 2021. The surveys included approximately 110 000 students, 14–17 years of age, and yielded a response rate varying from 95% in 1996 to 64% in 2021. A self-report questionnaire was used to collect information on symptoms of depression and loneliness. Information on antidepressant and sleep medication prescription was retrieved from the Norwegian Prescription Database for the age group 15 to 19 years. A graphical approach and logistic regression models were used to examine gender-specific time-trends between 1996 to 2021.

**Results:**

We found a doubling in self-reported symptoms of depression and loneliness among girls between 1996 and 2021, with the steepest increase in the period from 2006 to 2012, when Facebook and other social media were introduced. A similar trend was observed in the prescription of antidepressants among girls, with the steepest increase between 2011 and 2013. Among both boys and girls, ‘worried too much about things’ and ‘had sleep problems’ were the two symptoms with the greatest changes.

**Conclusion:**

A significant upward trend in self-reported depressive symptoms and medication use was observed over the past 25 years, with variations in the rate of increase, including a steeper trajectory during certain periods immediately after the introduction of social media platforms in Norway.

## Introduction

Several studies have reported increasing trends in symptoms of depression among adolescents, especially girls [1–3]. Results from the European Social Survey have shown an increase in depressive symptoms among young people aged 14–24 years in Spain, Norway and Denmark, especially in certain individual depressive symptoms such as fatigue, loneliness, and sleep problems [4]. Moreover, a study of Icelandic adolescents found that the proportion of those reporting a high degree of depressive symptoms rose by 1.6% for boys and 6.8% for girls, while for those reporting high anxiety symptoms, the increases were 1.3% for boys and 8.6% for girls [5]. Researchers studying trends in depressive symptoms in 16- to 17-year-old Norwegian adolescents at the three time points 1992, 2002, and 2010 revealed a significant increase in high scores on depressive symptoms between 1992 and 2002 in both boys and girls, but no increase between 2002 and 2010 [6]. However, between 2008 and 2017, a sharp rise in Norwegian adolescents’ use of prescription drugs for mental health problems has been reported, with a 60% and 18% rise in antidepressants among girls and boys, respectively [7]. Researchers have identified several determinants that might explain the increase in depressive symptoms in adolescents, although the picture is not entirely clear. A study reanalyzing previously collected data observed a consistent and substantial association between social media use and poorer mental health among girls, which was even stronger than the associations between poorer mental health and binge drinking, sexual assault, obesity, and drug use [8]. Further, according to the results from a recent study, more frequent use of electronic media was positively associated with depressive symptoms among Norwegian adolescents [9]. Loneliness has also been shown to be a risk factor for mental health problems. A large multi-national survey found that between 2012 and 2018, loneliness in schools increased in 36 out of the 37 countries studied [10]. Compared to 2012, nearly twice as many adolescents in 2018 had elevated levels of school loneliness, and the increase was higher among girls and in countries where smartphone use and internet access was high [10]. Sleep problems also count as depressive symptoms and contribute to poor mental health [11]. A cross-sectional study from the UK showed that poor sleep quality and short total sleep time at the age of 15 were predictive of anxiety and depression diagnoses at the ages of 17, 21, and 24, and, conversely, that depression at the age of 15 predicted less total sleep at 17 and 24 years of age [12]. Corresponding to this, the use of hypnotic drugs increased among young people in Scandinavia during 2012–2018, and the increase was twice as high in Sweden as in Denmark and Norway [13].

To summarize, research has shown increased mental health problems and use of prescription hypnotics and sedatives, and drugs for depression and anxiety among adolescents in recent years, and has also found a link between social media use and poorer mental health [14]. However, most longitudinal studies have focused on relatively short time intervals. To our knowledge, there are no studies using an ecological approach to investigate time trends in mental health problems and the prescription of drugs in relation to the emergence of social media. Thus, the aim of this study was to examine gender-specific trends in self-reported symptoms of depression and prescription of antidepressants, hypnotics and sedatives among Norwegian adolescents from 1996 to 2021. Further, we aimed to study how these trends align with loneliness and the introduction of social media during the same period.

## Methods

In this analysis, we used data from the youth survey ‘Ung i Oslo’ (Young in Oslo), available on demand from Norwegian Social Research (NOVA) and publicly accessible data from the Norwegian Prescription Database (NorPD), which we studied in relation to the emergence of different social media in Norway.

### Data sources

Young in Oslo is a survey of youth in Oslo, which in turn is part of ‘Ungdata’ [15, 16] a nationwide data collection scheme designed to conduct surveys among adolescents in Norway at the municipal level. Ungdata is regarded as the most comprehensive source of information on adolescent health, lifestyle, and well-being in Norway. The survey was initiated in 1996 and has thereafter been conducted at different time intervals (1996, 2006, 2012, 2015, 2018 and 2021) among lower secondary (about 13–16 years) and upper secondary (about 16–19 years) students in Oslo. Data were collected via paper questionnaires in the years 1996 and 2006, and electronically from 2012 onwards. Since 2010, the survey has also been conducted at the national level. To ensure data continuity over time, the present study only included data from the municipality of Oslo and adolescents in the final two years of lower secondary and the first year of upper secondary school (around 14–17 years). As participation in the surveys is voluntary, students may skip questions, and hence the number of responses varies between the variables. The Ungdata survey has been approved by the Norwegian Centre for Research Data (NSD) and by the Data Protection Office of Inland Hospital Trust (reference number 18778329). Data are collected anonymously, and do not contain information that might identify the participants. Therefore, no additional ethical approval was needed. The response rates were high in most of the six surveys conducted in the municipality of Oslo (in 1996 around 95%, 2006 93%, 2012 72%, 2015 79%, 2018 83%, and 2021 64%). The response rates were demographically comparable to Ungdata samples at the national level and thus considered representative of Norwegian adolescents [17–19]. A summary of the data is presented in Table 1.

**Table 1:** Sample characteristics by year of data collection from the Young in Oslo survey and the Norwegian prescription database.

Year	1996	2006	2012	2015	2018	2021
	**N (%)**					
**Gender**						
Male	5680 (51.0%)	5528 (48.8%)	4801 (49.2%)	11854(49.3%)	6412 (48.8%)	5065 (49.4%)
Female	5454 (49.0%)	5793 (51.2%)	4952 (50.8%)	12182 (50.7%)	6732 (51.2%)	5193(50.6%)
**Grade**						
9^th^ grade lower secondary school	3639 (32.6%)	3687 (32.3%)	3273 (32.9%)	8590 (34.2%)	4640 (35.2%)	3648 (34.7%)
10^th^ grade lower secondary school	3501 (31.3%)	3449 (30.3%)	3338 (33.6%	7764 (30.9%)	4205 (31.9%)	3727 (35.4%)
1^st^ grade upper secondary school	4039 (36.1%)	4262 (37.4%)	3323 (33.5%)	8790 (35.0%)	4348 (33.0%)	3142 (29.9%)
**HSCL-6 (Depressive symptoms)**						
Felt that everything is a struggle						
Low level	6543 (60.9%)	7030 (64.2%)	5427 (57.1%)	6828 (59.1%)	7120 (58.7%)	5419 (57.8%)
High level	4205 (39.1%)	3918 (35.8%)	4075 (42.9%)	4730 (40.9%)	5007 (41.3%)	3958 (42.2%)
Had sleep problems						
Low level	8431 (78.1%)	8294 (75.6%)	6172 (64.9%)	7904 (68.2%)	8019 (65.9%)	6172 (65.4%)
High level	2363 (21.9%)	2677 (24.4%)	3336 (35.1%)	3693 (31.8%)	4152 (34.1%)	3267 (34.6%)
Felt unhappy, sad or depressed						
Low level	8514 (79.0%)	8609 (78.7%)	7004 (73.8%)	8637 (74.7%)	8585 (70.9%)	6435 (68.4%)
High level	2262 (21.0%)	2332 (21.3%)	2486 (26.2%)	2927 (25.3%)	3530 (29.1%)	2977 (31.6%)
Felt hopelessness about the future						
Low level	8860 (82.5%)	8503 (78.0%)	7014 (74.1%)	8285 (71.7%)	8418 (69.6%)	6386 (68.0%)
High level	1876 (17.5%)	2401 (22.0%)	2455 (25.9%)	3273 (28.3%)	3685 (30.4%)	3004 (32.0%)
Felt stiff or tense						
Low level	8089 (75.6%)	8196 (75.5%)	6787 (71.9%)	8691 (75.5%)	8681 (72.1%)	6499 (69.7%)
High level	2610 (24.4%)	2658 (24.5%)	2648 (28.1%)	2820 (24.5%)	3355 (27.9%)	2826 (30.3%)
Worried too much about things						
Low level	6916 (64.5%)	6874 (63.0%)	5406 (57.1%)	382 (55.2%)	6133 (50.7%)	4622 (49.3%)
High level	3812 (35.5%)	4036 (37.0%)	4067 (42.9%)	5174 (44.8%)	5973 (49.3%)	4755 (50.7%)
**Loneliness**			Missing data			
Low level	6340 (94.1%)	9288 (90.8%)		8942 (79.5%)	9218 (76.2%)	6919 (73.6%)
High level	401 (5.9%)	936 (9.2%)		2306 (20.5%)	2886 (23.8%)	2482 (26.4%)
**Prescribed drugs year**	**2004**	**2006**	**2012**	**2015**	**2018**	**2020**
	**Users per 1000 inhabitants**					
**Antidepressants**						
Girls	13	13	18	9	11	10
Boys	7	7	7	21	23	24
**Hypnotics and sedatives**						
Girls	9	10	18	22	22	30
Boys	7	8	14	16	16	23

Further, we used data extracted from the NorPD, administered by the Norwegian Institute of Public Health, which provides aggregated data at the national level. The NorPD includes data on drugs dispensed throughout Norway between 2004 and 2020; however, it does not monitor drugs purchased without prescription (over the counter) or supplied to hospitals and nursing homes. The NorPD data are based on prescriptions to individuals registered in the NorPD with a valid national ID number. In 2020, the proportion of prescriptions having an invalid or missing national ID number was 0.10%, but this proportion was higher when the NorPD was established in 2004. In general, the proportion is higher among the youngest children.

Information on the emergence of different social media was gained from the Institut Publique de Sondage d’Opinion Secteur (IPSOS), an international market research and consulting firm with an office in Oslo, Norway (https://www.ipsos.com/nb-no). IPSOS publishes a quarterly social media user tracker, presenting data on the most frequently used social media in Norway, which are Facebook, Snapchat, Instagram, YouTube and TikTok [20]. From 2007 onwards, Facebook became available in Norwegian and replaced existing national social media, such as Nettby (2006–2009) and Blink (2002–2011) [21]. The video platform YouTube was established in 2005 and became popular in Norway around 2006/2007. From about 2012, the platform was available in Norwegian. Instagram, a photo and video sharing social networking service was established in 2010, followed by Snapchat, a mobile messaging application used to share photos, videos, text, and drawings, which was introduced in Norway in 2013. In 2018 TikTok, a Chinese short-form video hosting service became available worldwide after merging with another Chinese social media service [20]. Further, in 2020 about 90% of Norwegians aged 9–18 were active on social media; more specifically, 91% used YouTube, 80% Snapchat, 65% TikTok and 51% Facebook [22]. Thus, the emergence of the various social media platforms may be used as a proxy for their utilization within an ecological approach.

### Measures

#### Self-report of depressive symptoms

Self-reported depressive symptoms were measured using a six-item scale based on the Hopkins Symptom Checklist [23, 24]. The adolescents were asked if they had been affected by any of the following during the past week: ‘Felt that everything is a struggle’ (item 1), ‘had sleep problems’ (item 2), ‘felt unhappy, sad or depressed’ (item 3), ‘felt hopelessness about the future’ (item 4), ‘felt stiff or tense’ (item 5), ‘worried too much about things’ (item 6). The six questions have four response categories: 1 = ‘not been affected at all’, 2 = ‘not been affected much’, 3 = ‘been affected quite a lot’, and 4 = ‘been affected a great deal’. The six-item scale has been psychometrically evaluated among Norwegian adolescents. It has been shown to have good reliability (Person Separation Index: 0.802), and as a whole, the scale works reasonably well at a general level [25]. Usually, sum scores are computed, ranging from 6 to 24, where high scores indicate higher symptoms of depression. In the current study, we present both the sum score and the six items as single-item variables. In the latter case, variables were dichotomized into 0 = low level (‘Not been affected at all’ and ‘not been affected much’) and 1 = high level (‘been affected quite a lot’ and ‘been affected a great deal’) of the different symptoms of depression [26]. We used the same procedure for the variable loneliness, which was assessed by the question ‘Did you feel lonely in the last seven days?’ with the four response options mentioned above [27]. Data on loneliness in the year 2012 was missing.

In the surveys conducted in 1996 and 2006, the response options of the items assessing symptoms of depression and loneliness were in the reverse order from that presented above. This also applied to the survey in 2012, where half of the participants were asked questions with a reverse response order [28]. For statistical reasons, the response order in the surveys in 1996, 2006, and 2012 was re-coded in this study. Previous research has shown that the effect of the order is moderate, indicating that it is primarily other factors that affect how young people respond [28].

#### Antidepressants, hypnotics, and sedatives

Antidepressants were identified by the ATC code N06A, and hypnotics and sedatives by the ATC code N05C, including N05CH01 melatonin. Prevalence was calculated as the number of users per 1000 inhabitants per year. A *user* was defined as a person who has had at least one prescription for either antidepressants or hypnotics and sedatives dispensed in a pharmacy during the period. A person who has had several prescriptions for the same drug during the same year is only counted once. We extracted the number of users per year for adolescents residing in Oslo aged 15–19 years, as publicly available data from the NorPD only provides data in five-year age groups.

### Statistical analysis

We first used graphical approaches and descriptive statistics to identify patterns in symptoms of depression and prescription of antidepressants, hypnotics and sedatives in relation to the emergence of different social media. After testing the data on symptoms of depression for linearity, we proceeded with logistic regression to analyze the trends in prevalence of high level of depressive symptoms over time. The dependent variable was symptoms of depression (high level = 1; no symptom/low level = 0) constructed by the sum-score of the 6 items measuring symptoms of depression, and the categorical predictor variables were year (1996 as the reference) and gender (male as a reference category). In a second step, the analysis was stratified by gender. We then matched data from the NorPD with Young in Oslo by year and gender and then computed pairwise Pearson correlation coefficients between the six variables for symptoms of depression and the prescription of antidepressants, hypnotics and sedatives, separately for boys and girls. Data were normally distributed among girls and almost normally distributed among boys. Statistical significance was set at p <0.05. All analyses were conducted using IBM SPSS Statistics for Windows (IBM Corp, Version 26.0, released 2016, Armonk, NY).

## Results

### Self-reported symptoms of depression and loneliness

**Fig 1:**
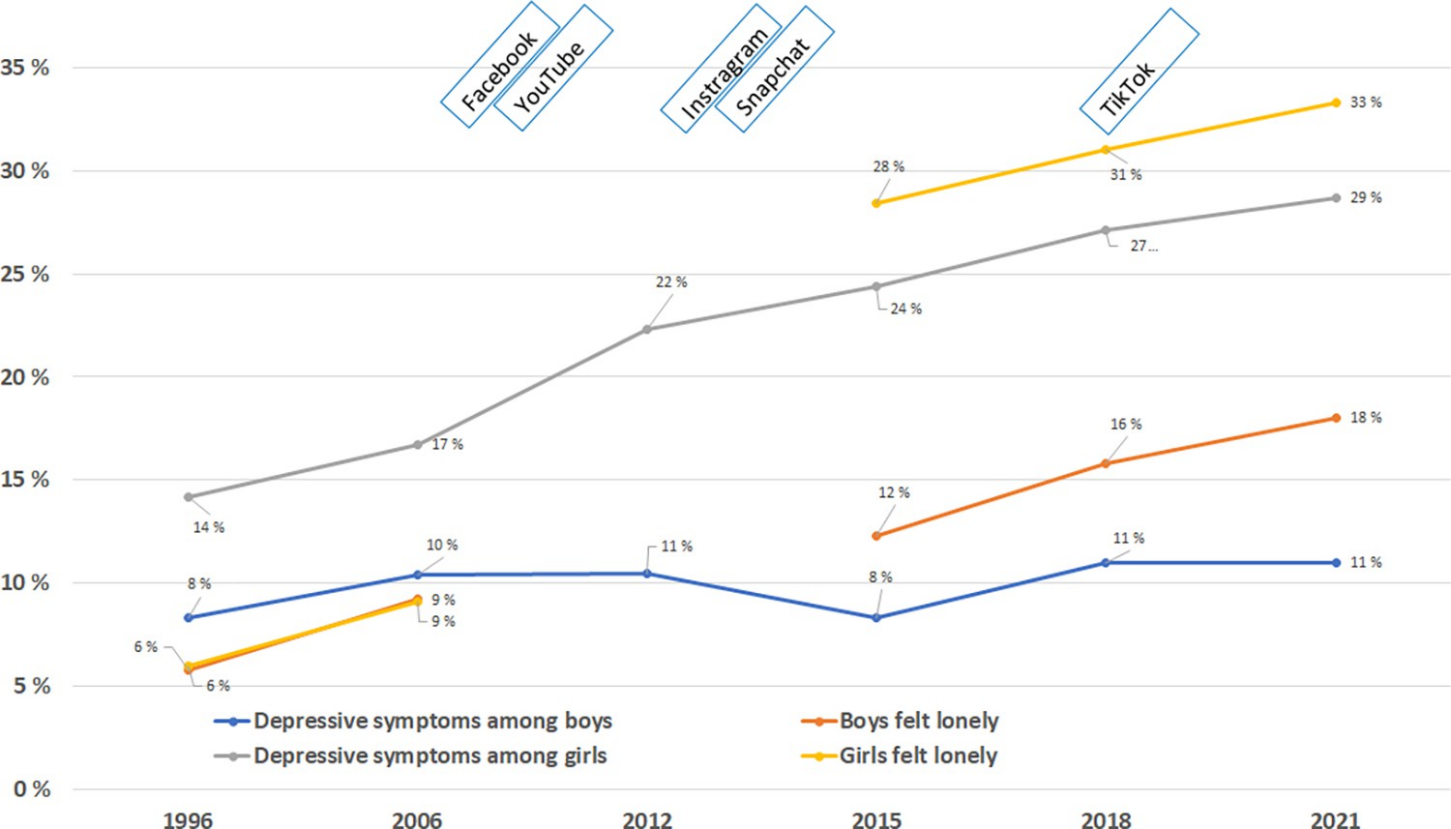
Trends in self-reported depressive symptoms (sum score) and loneliness among girls and boys (Fig 1) aged 14–17 years and the emergence of social media from 1996 to 2021. The lines show the percentage of participants with high-level depressive symptoms and loneliness over time.

**Fig 2:**
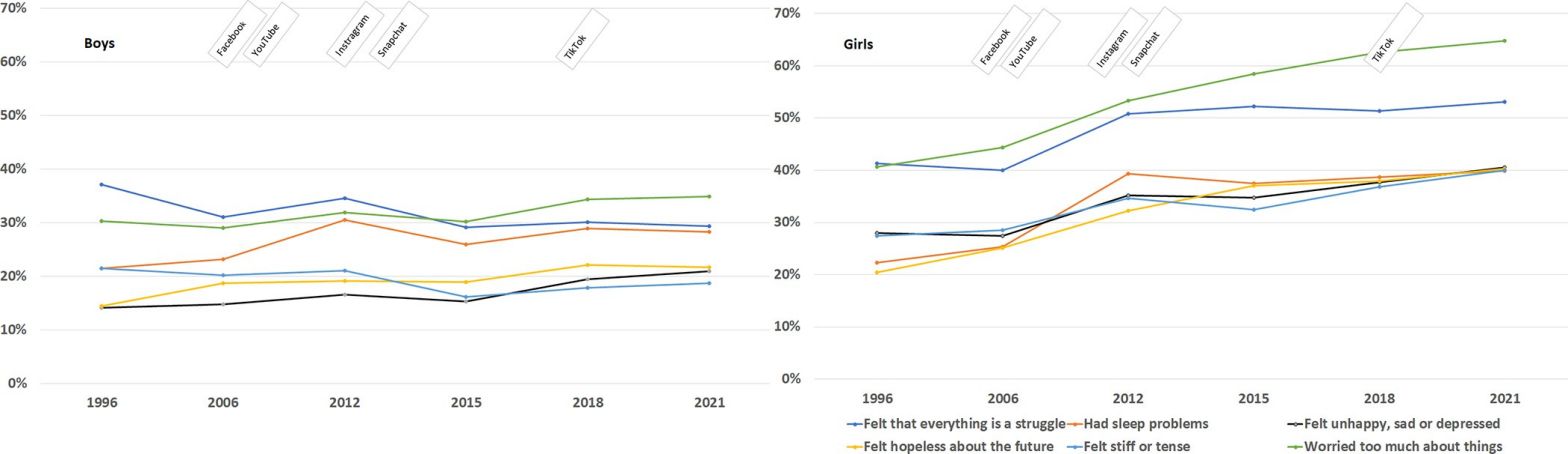
Trends in six self-reported depressive symptoms (derived from the Hopkins Symptom Checklist) among girls and boys (Fig 2) aged 14–17 years and the emergence of social media from 1996 to 2021.

Among adolescents aged 14–17 years, the findings showed an increase in self-reported symptoms of depression from 1996 to 2021, and girls were generally more affected than boys (Fig 1). The percentage of girls affected by symptoms of depression doubled from 1996 (14.2%) to 2021 (28.7%), with the greatest increase between 2006 (16.7%) and 2012 (22.3%). High level of self-reported depressive symptoms was associated with female gender (OR 2.6 (CI: 2.5–2.7), see Table 2. In the gender-specific time trend analysis, we observed increased prevalence of depressive symptoms among girls, but not boys, which was interrupted in the year 2015 (Table 2 and Fig 1). The percentage of girls who felt lonely increased from 6% in 1996 to 33.3% in 2021, with the greatest increase between 2006 (9.1%) and 2015 (28.4%), although data from 2012 were missing (Fig 1). The regression model confirmed a statistically significant increase in loneliness over time among girls (and boys. Overall, girls (OR 2.0 (CI: 1.9–2.1) were 2 times more likely to have felt lonely than boys (Table 2). Considering each of the six depressive symptom items separately, most of the study participants, regardless of gender, reported an increase in ‘had sleep problems’ between 2006 and 2012 (Fig 2). However, the most prevalent depressive symptom was ‘worried too much about things’, in both boys (34.9%) and girls (64.8%). The trend for the symptom ‘worried too much about things’, was almost parallel with the trend for the symptom ‘feeling hopelessness about the future’, with a continuous difference of 20% in girls and 12% in boys over the entire study period, 2006–2021 (Fig 2). While there was no distinct change pattern among boys regarding the six single items, scores on all six single items increased continuously among girls, although the trend flattened out from 2018 onwards. The steepest increase in all six self-reported symptoms of depression in both genders was seen between 2006 and 2012, the period of the emergence of Facebook, YouTube, Instagram and Snapchat.

**Table 2:** Logistic regression models for self-reported depressive symptoms and loneliness as the dependent variables.

Variable	Depressive symptoms			Loneliness		
		Girls	Boys		Girls	Boys
	Odd ratio (95% CI) ^a^	Odd ratio (95% CI) ^b^	Odd ratio (95% CI) ^b^	Odd ratio (95% CI) ^a^	Odd ratio (95% CI) ^b^	Odd ratio (95% CI) ^b^
**Gender** (ref. boy)						
Girl	2.6 (2.5–2.7) ***	n.a.	n.a.	2.0 (1.9–2.1) ***	n.a.	n.a.
**Year**						
1996	1	1	1	1	1	1
2006	1.2 (1.1–1.3) ***	1.2(1.1–1.3) ***	1.3(1.1–1.1) ***	1.6 (1.4–1.8) ***	1.6(1.3–1.9) ***	1.6(1.4–2.2) ***
2012	1.6 (1.4–1.7) ***	1.7(1.6–1.9) ***	1.3(1.1–1.5) ***	n.a.	n.a.	n.a.
2015	1.6 (1.4–1.7) ***	1.9(1.8–2.2) ***	1.0(0.9–1.2)	4.1 (3.7–4.6) ***	6.2(5.4–7.2) ***	2.3(1.9–2.7) ***
2018	1.9(1.8–2.2) ***	2.2(2.0–2.5) ***	1.4(1.2–1.6) ***	5.0(4.4–5.5) ***	7.0(6.1–8.1) ***	3.0(2.6–3.6) ***
2021	2.0(1.8–2.2) ***	2.4(2.2–2.7) ***	1.4(1.1–1.5) ***	5.7(5.1–6.3) ***	7.8(6.7–9.1) ***	3.5(3.0.1–4.2) ***

n.a.: not applicable

CI: Confidence interval

***: p≤ 0.001

^a^ Multivariate logistic regression adjusted for gender and year.

^b^ Logistic regression models for girls and boys respectively

Trends in depressive symptoms and loneliness in relation to the emergence of different social media are visualized in Figs 1 and 2.

### Prescription of antidepressants, hypnotics and sedatives

**Fig 3:**
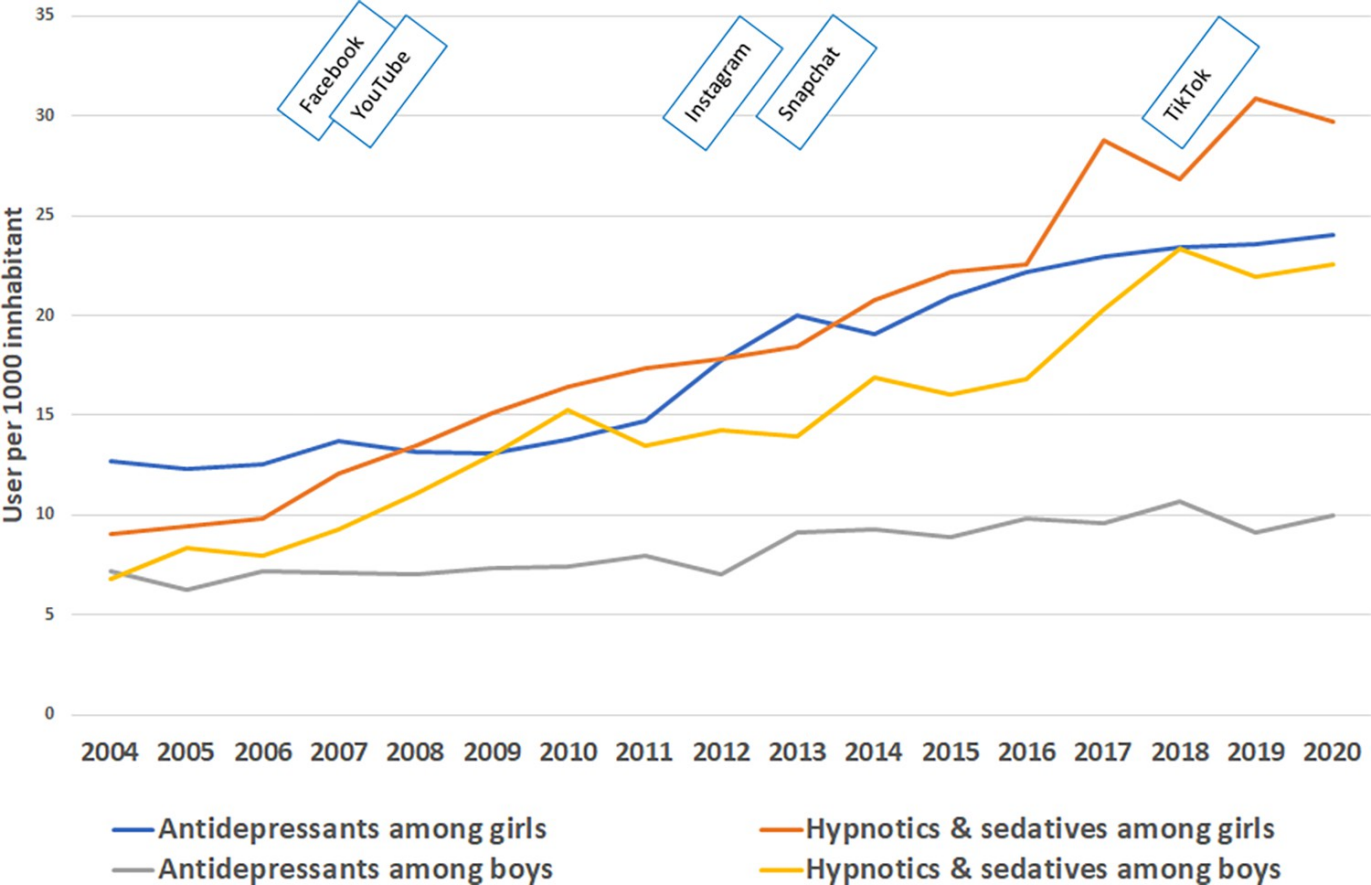
Prevalence of use of antidepressants and hypnotics and sedatives among girls and boys (Fig 3) aged 15–19 years and the emergence of social media from 2004–2020.

The prevalence of the prescription of antidepressants, hypnotics and sedatives by gender is shown in Fig 3. Analogous to the rise in depressive symptoms in adolescents, the findings showed an increase in the prescription of antidepressants, hypnotics and sedatives per 1000 inhabitants between 2004 and 2020. While the prevalence of prescribed antidepressants among boys was quite constant over the years, a steep rise in prevalence in girls was seen between 2006 and 2012. The curve showed the highest prevalence for the prescription of hypnotics and sedatives among girls in 2019 and among boys in 2018. The increase was steepest between 2016 and 2018 in both genders.

### Correlations between depressive symptoms and prescription of drugs

We used pairwise correlation coefficients to quantify the relationships between the six depressive symptoms and the prescription of antidepressants, hypnotics and sedatives across the two data sources (Table 3A and 3B). Among girls (Table 3A), the measures tended to correlate very highly with each other. For instance, ‘worried too much about things’ correlated very highly with the prescription of both antidepressants (r = 1.00, p<0.001) and hypnotics and sedatives (r = 0.99, p = 0.001). A similar correlation, was seen between the measure ‘felt hopelessness about the future’ and the prescription of antidepressants and hypnotics/sedatives, respectively (r = 0.99, p = 0.013; r = 0.98, p = 0.022). There was no statistically significant correlation between ‘had sleep problems’ and the prescription of hypnotics and sedatives in either gender. Among boys (Table 3B), the picture looks slightly different. There were no statistically significant correlations between the prescription of antidepressants and any of the six depressive symptoms. ‘Felt unhappy, sad or depressed’ was however correlated with ‘felt hopelessness about the future’ (r = 0.86, p = 0.027) and ‘worried too much about things’ (r = 0.96, p = 0.002). Further, there was a positive correlation between ‘felt stiff or tense’ and ‘felt everything is a struggle’ (r = 0.86, p = 0.029). The prescription of hypnotics and sedatives correlated highly with the prescription of antidepressants in both genders (r = 0.946, p<0.001 for girls and r = 0.863, p<0.001 for boys).

**Table 3:** Pairwise pearson correlation coefficients (p-value) between antidepressants, hypnotics and sedatives, and the six measures of depressive symptoms derived from the Hopkins Symptom Checklist. Note: Data from Young in Oslo covers the years 1996, 2004, 2012, 2015, 2018, and 2021, while the prescription database covers the years 2004–2020.

**A. Girls**							
	**Hypnotics and sedatives**	**Felt that everything is a struggle**	**Had sleep problems**	**Felt unhappy, sad or depressed**	**Felt hopelessness about the future**	**Felt stiff or tense**	**Worried too much about things**
**Antidepressants**	0.946** (<0.001)	0.885 (0.115)	0.840 (0.160)	0.938 (0.062)	0.987* (0.013)	0.862 (0.138)	1.000** (<0.001)
**Hypnotics and sedatives**		0.874 (0.126)	0.837 (0.163)	0.942 (0.058)	0.978* (0.022)	0.879 (0.121)	0.999** (0.001)
**Felt that everything is a struggle**			0.969** (0.001)	0.946** (0.004)	0.938** (0.006)	0.874* (0.023)	0.929** (0.007)
**Had sleep problems**				0.932** (0.007)	0.938** (0.006)	0.896* (0.016)	0.921** (0.009)
**Felt unhappy, sad or depressed**					0.944** (0.005)	0.980** (<0.001)	0.965** (0.002)
**Felt hopelessness about the future**						0.910*(0.012)	0.992**(<0.001)
**Felt stiff or tense**							0.937** (0.006)
**B. Boys**							
	**Hypnotics and sedatives**	**Felt that everything is a struggle**	**Had sleep problems**	**Felt unhappy, sad or depressed**	**Felt hopelessness about the future**	**Felt stiff or tense**	**Worried too much about things**
**Antidepressants**	0.863** (<0.001)	-0.650 (0.350)	0.226 (0.774)	0.760 (0.240)	0.864 (0.136)	-0.715 (0.285)	0.713 (0.287)
**Hypnotics and sedatives**		-0.292 (0.708)	0.628 (0.372)	0.889 (0.111)	0.873 (0.127)	-0.519 (0.481)	0.907 (0.093)
**Felt that everything is a struggle**			-0.345 (0.503)	-0.544 (0.265)	-0.792 (0.060)	0.856* (0.029)	-0.346 (0.502)
**Had sleep problems**				0.711 (0.113)	0.763 (0.077)	-0.263 (0. .615)	0.710 (0.114)
**Felt unhappy, sad or depressed**					0.863* (0.027)	-0.346 (0. .502)	0.961** (0.002)
**Felt hopelessness about the future**						-0.562 (0. .246)	0.738 (0.094)
**Felt stiff or tense**							-0.252 (0.631)

*p<0.05

**p<0.1 (0.006)

*p<0.05, **p<0.1

## Discussion

### Main summary of results

This trend analysis shows a doubling of self-reported high-level depressive symptoms in girls aged 14–17 between 1996 and 2021. The largest increase in depressive symptoms was observed between 2006 and 2012, which was the period when Facebook and other social media were introduced in Norway. This trend was followed by an increase in the prescription of antidepressants, with the steepest increase from 2011 to 2013. Among boys and girls alike, the most prevalent depressive symptom between 1996 and 2021 was “worried too much about things”.

### Time trends in depressive symptoms and drug prescriptions

Several studies from Norway and other countries have described similar trends linking an increase in symptoms of depression to the introduction of social media [1, 29, 30]. A study among Norwegian adolescents and young adults reported a sharp increase in mental distress over time, especially during the periods 2006–2008 and 2017–2019. The authors pointed to the technology industry’s strong influence on young people’s behavior and stated that their use of manipulative and exploitative strategies may be an important driver of the recent increase in young people’s mental health challenges [31].

However, the picture is not entirely clear. Previous research suggests that both excessive social media use and no social media use at all are both associated with depressive symptoms, whereas moderate social media use seems to reduce the risk of depressive symptoms [32, 33]. One explanation might be that adolescents who do not use social media at all lack access to an important source of inclusion and belonging [34]. On the other hand, adolescents using social media excessively may be more inclined to experience lack of control and social media addiction [35], problematic comparisons with peers [36, 37], or negative events, such as online bullying and harassment [38]. Thus, abstinence from, as well as excessive use of, social media may have consequences in the form of depressive symptoms. Another explanation for the increase in self-reported depressive symptoms over the past 25 years may be the increased openness and ability among adolescents to put their distress and mental health challenges into words [39]. Social media may also promote this, where it is easy to get in touch, optionally anonymously, with peers who might suffer from the same challenges [40].

A new insight from this study is that increases in self-reported depressive symptoms, especially in girls, were followed by a trend of increased prescription of antidepressants and hypnotics and sedatives. The steepest increase in the prescription of antidepressants seemed to be from 2011 to 2013. There might be several reasons why the largest increase in prescription of antidepressants occurred somewhat later than the steepest increase in self-reported depressive symptoms. One possible explanation is that the prescription peak is artificial as the prescription data also include adolescents up to the age of 19 years, compared to 14–17 years in the Young in Oslo surveys. There might also be a backlog effect from the first symptoms to accessing health care services.

Another new insight from this study is that worry seems to be the main driver of the increase in depressive symptoms. In line with our study, previous research has shown that females worry more about things than males [41]. This increase in self-reported depressive symptoms in girls but not boys might be explained by the substantially increasing scores on the item ‘worried too much about things’ among girls across the whole study period, with a steeper increase from 2006 onwards, while scores among boys were relatively stable. Moreover, previous research has reported that the ‘worry’ item seems to work differently for males and females [25]. Thus, girls appear to be more inclined to worry than boys are, and further, the emergence of social media may have given girls more to worry about. The inherent pitfalls of social media in terms of social comparison and the presentation of an ideal, false self may affect girls to a greater degree than boys [42, 43]. Worry might come from different sources, like being worried about present problems, such as being good enough and having friends, but one can also worry about the future. Our analysis showed that ‘feeling hopelessness about the future’ followed the same trend as ‘worried too much about things’. In a recent study, we found that 40% of the adolescents worried about climate change [44], which might be an indicator that worry in general and negative thoughts about the future go hand in hand. Another explanation for worries in adolescents in recent years might be the unstable socio-economic situation following the global financial crisis of 2008 [45]. However, the unemployment rate in Norway is among the lowest in the world [46], and the financial crisis does not seem to have had a major impact on the mental health of the Norwegian population in general [47].

It is, however, important to exercise caution in interpreting the results, as the responses in the survey do not necessarily mean that participants have been diagnosed with depression, as our analysis cannot tell us if participants’ actual mental health diagnosis became poorer. Proxy markers might be misleading in mental health and interpretation should be vetted carefully. Trends in prescription data might also have other underlying reasons than a true increase in depression and sleep problems, such as off-label use, wider indications for use and changes in physicians’ attitude to prescription.

In the present study, we also found an increase in the proportion of adolescents feeling lonely and a concurrent increase in use of hypnotics and sedatives across a 25-year period. However, the increase of loneliness may also be due to the shift in leisure activities in recent years, from group activities like dancing, choirs, bands, and theater to more individual activities such as watching films or chatting on a mobile [48, 49]. This leisure trend might also have been reinforced by the removal of public open spaces for adolescents, leaving less opportunity to meet peers [50]. In addition, a study by Matthews et al. found that loneliness was robustly associated with poorer sleep quality in young people, which might explain the increase in prescribed sleep medication [51]. Other research links social media use to a tendency among adolescents to stay awake and alert during late evenings and nights [52].

Of the items measuring self-reported depressive symptoms, the steepest increase in the item ‘having sleep problems’ was seen between 2006 and 2012. This increase could be observed in both genders. Previous studies have discussed problems with the interpretation of this item, as the wording is very imprecise [25]. Sleep problems might be problems with falling asleep, staying asleep, waking up, or restless sleep [53] In addition, this item is used as part of a scale intended to measure depressive symptoms, but a previous study has emphasized that ‘sleep problems’ are not necessarily related to depressive symptoms. Reporting sleep problems may alternatively be due to adolescents’ screen time late at night, leading to insufficient sleep [25]. This might be one possible explanation of the steep increase in ‘sleep problems’ from 2006–2012, the period when several social media emerged and became popular in Norway. While sleep problems may not necessarily be a depressive symptom, poor sleep quality or insufficient sleep is a risk factor for mental health problems [54].

There are further reasons to interpret the results with caution. First, the analysis is based on self-reported depressive symptoms, which might not reflect the actual burden of depression. Second, national surveys from the years 2020–2022 show that adolescents report generally high subjective well-being [16, 55, 56], although they do indicate being affected by the various depressive symptoms. This may be due to a growing openness to expressing different kinds of depressive symptoms. Since 2020, the subject ‘Public Health and Life Mastery’ has been implemented in secondary schools. A study conducted in Norway showed that teaching secondary school students about mental health did not decrease depressive symptoms. On the contrary, the adolescents showed a higher level of depressive symptoms after 25 weeks of teaching about mental health [57].

There has also been speculation about the possible normalization of mental health problems among adolescents, and the use of medical terms to describe what were previously considered as normal life challenges [58]. Ultimately, we might medicalize a whole generation of youth and thereby create more illness and less resilience, also by publishing research on increasing trends in illness that may or may not be connected to real illness [59].

### Study strengths and limitations

The main strength of the study is the large and representative sample of Norwegian adolescents and the use of self-reported validated questions. In addition, we used multiple sources of information, merging cross-sectional personal data with aggregated data from the prescription register in different periods (despite no prescription data from 1996 and 2004).

There are also some important limitations. First, we are unable to address causality due to the ecological design. We also had to change the order of the response categories in the six items measuring depressive symptoms from 2012, which might have led to artificially high scores on depressive symptoms in that period. Another limitation in the interpretation of the results is that the age groups are categorized differently between the two data sources. One could argue that time spent on social media would have been a more precise measure than the emergence of several social media platforms, but this would have limited the observation period as data on time spent on social media is not available for the time frame of the study (1996–2021).

## Conclusion and implications

We observed an upward trend in self-reported depressive symptoms and the prescription of antidepressants and hypnotics and sedatives over the past 25 years, with variations in the rate of increase, including a steeper trajectory during certain periods immediately after the introduction of social media platforms in Norway. Due to the ecological design of this study, causal conclusions are difficult to draw, but the results shed light on the possible impact of social media in apparently increasing depressive symptoms in adolescents, particularly girls, by giving rise to more worry and more sleep problems. The main implication of the study is that adolescents need to learn more about the possible negative influence of social media use on mental health.

## Supporting information

S1 Data (XLSX)
