# Correction: Time trends in self-reported depressive symptoms, prescription of antidepressants, sedatives and hypnotics and the emergence of social media among Norwegian adolescents

**DOI:** 10.1371/journal.pone.0306972

**Published:** 2024-07-05

**Authors:** Lars Lien, Tore Bonsaksen, Tonje Holte Stea, Annette Løvheim Kleppang, Anne Mari Steigen, Marja Leonhardt

In Table 1, the numbers under the “Antidepressants” of User per 1000 inhabitants for the years 2015, 2018 and 2021 are interchanged. It should have been 21, 23 and 24 for girls and 9, 11, 10 for boys respectively. Please see the correct Table 1 here.

**Table 1:** Sample characteristics by year of data collection from the Young in Oslo survey and the Norwegian Prescription Database

Year	1996	2006	2012	2015	2018	2021
	**N (%)**					
**Gender** Male Female	5680 (51.0%) 5454 (49.0%)	5528 (48.8%) 5793 (51.2%)	4801 (49.2%) 4952 (50.8%)	11854(49.3%) 12182 (50.7%)	6412 (48.8%) 6732 (51.2%)	5065 (49.4%) 5193(50.6%)
**Grade** 9^th^ grade lower secondary school 10^th^ grade lower secondary school 1^st^ grade upper secondary school	3639 (32.6%) 3501 (31.3%) 4039 (36.1%)	3687 (32.3%) 3449 (30.3%) 4262 (37.4%)	3273 (32.9%) 3338 (33.6% 3323 (33.5%)	8590 (34.2%) 7764 (30.9%) 8790 (35.0%)	4640 (35.2%) 4205 (31.9%) 4348 (33.0%)	3648 (34.7%) 3727 (35.4%) 3142 (29.9%)
**HSCL-6 (Depressive symptoms)** Felt that everything is a struggle Low level High level	6543 (60.9%) 4205 (39.1%)	7030 (64.2%) 3918 (35.8%)	5427 (57.1%) 4075 (42.9%)	6828 (59.1%) 4730 (40.9%)	7120 (58.7%) 5007 (41.3%)	5419 (57.8%) 3958 (42.2%)
Had sleep problems Low level High level	8431 (78.1%) 2363 (21.9%)	8294 (75.6%) 2677 (24.4%)	6172 (64.9%) 3336 (35.1%)	7904 (68.2%) 3693 (31.8%)	8019 (65.9%) 4152 (34.1%)	6172 (65.4%) 3267 (34.6%)
Felt unhappy, sad or depressed Low level High level	8514 (79.0%) 2262 (21.0%)	8609 (78.7%) 2332 (21.3%)	7004 (73.8%) 2486 (26.2%)	8637 (74.7%) 2927 (25.3%)	8585 (70.9%) 3530 (29.1%)	6435 (68.4%) 2977 (31.6%)
Felt hopelessness about the future Low level High level	8860 (82.5%) 1876 (17.5%)	8503 (78.0%) 2401 (22.0%)	7014 (74.1%) 2455 (25.9%)	8285 (71.7%) 3273 (28.3%)	8418 (69.6%) 3685 (30.4%)	6386 (68.0%) 3004 (32.0%)
Felt stiff or tense Low level High level	8089 (75.6%) 2610 (24.4%)	8196 (75.5%) 2658 (24.5%)	6787 (71.9%) 2648 (28.1%)	8691 (75.5%) 2820 (24.5%)	8681 (72.1%) 3355 (27.9%)	6499 (69.7%) 2826 (30.3%)
Worried too much about things Low level High level	6916 (64.5%) 3812 (35.5%)	6874 (63.0%) 4036 (37.0%)	5406 (57.1%) 4067 (42.9%)	6382 (55.2%) 5174 (44.8%)	6133 (50.7%) 5973 (49.3%)	4622 (49.3%) 4755 (50.7%)
**Loneliness** Low level High level	6340 (94.1%) 401 (5.9%)	9288 (90.8%) 936 (9.2%)	Missing data	8942 (79.5%) 2306 (20.5%)	9218 (76.2%) 2886 (23.8%)	6919 (73.6%) 2482 (26.4%)
**Prescribed drugs year**	**2004**	**2006**	**2012**	**2015**	**2018**	**2020**
	**Users per 1000 inhabitants**					
**Antidepressants** Girls Boys	13 7	13 7	18 7	21 9	23 11	24 10
**Hypnotics and sedatives** Girls Boys	9 7	10 8	18 14	22 16	22 16	30 23
